# NOVA food groups’ consumption associated with nutrient intake profile of school children aged 8–12 years

**DOI:** 10.1017/S1368980022000441

**Published:** 2022-02-21

**Authors:** Arabele Teixeira de Lacerda, Ariene Silva do Carmo, Taciana Maia de Sousa, Luana Caroline dos Santos

**Affiliations:** Universidade Federal de Minas Gerais, Escola de Enfermagem, Departamento de Nutrição, Avenida Professor Alfredo Balena, 190, 3º Andar, Sala: 324, Santa Efigênia, 30130-100 Belo Horizonte, Minas Gerais 30130100, Brazil

**Keywords:** Industrialised food, Child nutrition, Dietary guidelines, Nutrients

## Abstract

**Objective:**

To evaluate the association between the consumption of NOVA food groups (classification based on the nature, extent and purpose of food processing) and the intake of energy, macro and micronutrients among school children.

**Design:**

Cross-sectional study. Food consumption was assessed by two 24-h dietary recalls on non-consecutive days. Energy from each NOVA food groups – ultra-processed foods, unprocessed or minimally processed foods, processed culinary ingredients and processed foods – was estimated. For analysis, the percentage of energy from ultra-processed foods and unprocessed or minimally processed foods were categorised into tertiles and associated with intake of energy, macro and micronutrients using analysis of covariance and linear regression.

**Setting:**

Public schools in Belo Horizonte, Minas Gerais, Brazil.

**Participants:**

School children aged 8–12 years (*n* 797; 406 girls; 391 boys).

**Results:**

Mean energy intake was 2050·18 ± 966·83 kcal/d, 25·8 % was from ultra-processed foods, 56·7 % from unprocessed or minimally processed foods, 8·9 % from processed culinary ingredients and 8·6 % from processed foods. A higher energy contribution from ultra-processed foods was negatively associated with the intake of protein, fibre, vitamin A, Fe and Zn (*P* < 0·001) and positively associated with total energy, lipid and Na intake (*P* < 0·001). Concurrently, a higher energy contribution from unprocessed or minimally processed foods was positively associated with the consumption of protein, fibre, Fe and Zn (*P* < 0·001) and negatively associated with total energy (*P* = 0·002), lipid and Na intake (*P* < 0·001).

**Conclusions:**

In conclusion, higher ultra-processed food consumption presented a negative association with the nutrient intake profile of school children.

Recently, the importance of food processing has increased due its potential role in diet-related non-communicable diseases^(1)^. Therefore, Monteiro *et al.* ^(2)^ proposed a new food classification according to the extent and purpose of food processing called NOVA. This classification is recognised by the FAO of the UN and the Pan American Health Organization as a valid tool for nutrition and public health research^(1,2)^.

NOVA separates food into four groups: unprocessed and minimally processed foods; processed culinary ingredients; processed foods and ultra-processed foods^(1,2)^. The Brazilian Dietary Guidelines follow this new classification by strongly recommending a diet based on unprocessed or minimally processed foods, moderate consumption of processed culinary ingredients and processed foods and avoidance of ultra-processed foods^(3)^. These recommendations are based on current scientific evidence and changes in the dietary pattern in Brazil and worldwide^(1–3)^.

A reduction in the consumption of unprocessed or minimally processed foods among school children in recent years, along with an increase in ultra-processed foods, have been described in national and international studies^(4,5)^. It is known that highly processed foods generally have greater energy density and higher fat, sugar and Na content compared with unprocessed or minimally processed foods. In addition, they are hyperpalatable and easily accessible foods, facilitating their consumption^(1,6,7)^.

Although school meals in Brazilian public schools are regulated by the National School Food Program (*Programa Nacional de Alimentação Escolar* – PNAE), which advocates the provision of healthy meals^(8)^, students have access to ultra-processed foods sold outside the school or in the home. The regular consumption of ultra-processed foods among school children may increase the risk of non-communicable chronic diseases such as obesity, diabetes, hypertension and dyslipidaemia and promote inadequacies in micronutrients intake^(9)^. Although nutritional deficiencies can be caused by non-dietary factors, insufficient intake of micronutrients is pointed out as the main cause^(10)^.

Previous studies with Brazilian children found a high prevalence of dietary inadequacy of various vitamins and minerals, mainly Fe, vitamin A and Zn^(8,11–13)^. According to a national survey, a high intake of ultra-processed foods had a negative impact on the dietary components of foods consumed by Brazilian individuals above 10 years of age^(14–16)^. However, few studies have focussed on children and adolescents are still incipient, which highlights the need to investigate the factors associated with the consumption of ultra-processed foods in this population.

Considering this scenario, the current study aimed to evaluate the association between NOVA food groups’ consumption and the intake of energy, macro and micronutrients among school children.

## Methods

### Study design and population

This is a cross-sectional study conducted between 2014 and 2015 with school children aged 8–12 years of public elementary schools located in a Brazilian metropolis (Belo Horizonte, 330·9 km^2^; 2 350 564 inhabitants)^(17)^.

The study sample was estimated considering 50 % for a given characteristic (in order to obtain the largest sample size), finite population (*n* 10 623), setting the significance level at 5 % (alpha or type I error) and sample error at 5 %, according to the criteria of Hulley and Cummings^(18)^. In addition, this value was doubled considering the two-stage cluster sampling (schools and classes) with proportionate stratification based on location. The value (*n* 742) was distributed proportionally to the size of each region (the nine regions of Belo Horizonte). From this distribution, seventeen schools were selected via simple cluster samples, stratified by the nine regions of the metropolis.

The seventeen schools selected had a total of 931 students in the mentioned grade (4th year), who were invited to participate in the research. Of the 931 students invited, children who were absent on the day of data collection (*n* 101), who refused to participate in the survey (*n* 2) or who had compromised mental health according to teachers’ report were not evaluated (*n* 31), and the final sample was 797 students. The school children excluded from the study were not statistically different in terms of sex, age and municipal region (*P* > 0·05).

### Data collection

Information regarding sex, date of birth and address of the children was obtained from school documents. Using the addresses of the children, the Health Vulnerability Index (HVI) of their residences was identified, which was used as a proxy of socio-economic status. HVI is a indicator that associates different socio-economic and environmental variables for the analysis of population characteristics in certain geographic areas^(19)^. This indicator is classified into four categories: low, medium, high and very high^(20)^.

Anthropometric variables (weight and height) and food consumption of the students were collected in person in their respective schools by a trained health professional. Anthropometric evaluation was performed according to the techniques recommended by the WHO^(21)^, and BMI [BMI = weight (kg)/height (meters) 2] -by-age was calculated. BMI-by-age was classified according to the cutoff points proposed by the Brazilian Food and Nutrition Surveillance System^(22)^ based on the WHO growth charts^(23)^. The school children were considered overweight when presented BMI-by-age values > z-score +1^(22)^.

Food consumption was assessed by two 24-h dietary recalls (24hR) on non-consecutive days, with a maximum interval of 7 days, including only school days. School children were interviewed by a trained dietitian or nutrition student, using the Automated Multiple-Pass Method^(24)^. During the 24hR, a list of illustrations of household measures was used to facilitate the identification of the real portion size consumed and improve information consistency.

The food consumption reported by the school children in household measures was transformed into grams or millilitre units, and its nutritional composition was evaluated according to information from Brazilian food composition tables using the software Stata^®^ version 11.

**Fig. 1:**
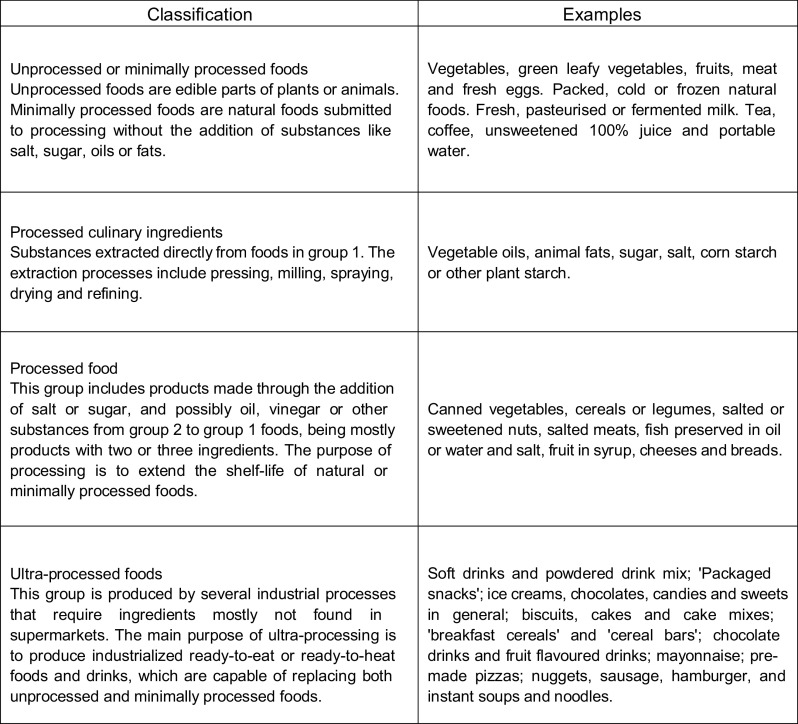
NOVA food groups: definition according to the extent and purpose of food processing, Adapted by Monteiro *et al.* (2016).

The items present in the food consumption were classified according to NOVA food groups based on the extent and purpose of food processing^(1)^ (Fig. 1). Ingredients from food dishes were classified separately.

The mean intake of energy (kJ), carbohydrates (% total energy value – TEV), proteins (%TEV) and lipids (%TEV) were evaluated, along with Na (mg), fibres (g) and micronutrients involved in child development: Ca (mg), Fe (mg), vitamin A (μg), vitamin C (mg) and Zn (mg)^(25)^. The content of each nutrient in diet was corrected by energy, being expressed per 4·18 MJ (1000 kcal).

Subsequently, the percentage contribution of ultra-processed foods to the TEV as well as percentage contribution of unprocessed or minimally processed foods (%TEV) was quantified. This percentage was categorised as tertiles which represented the strata distribution of food contribution according to food groups – ultra-processed or sum of the other three food categories – to the TEV.

### Statistical analysis

Frequency distributions, means and standard deviations were calculated. The normality was evaluated by the Shapiro–Wilk test. Differences between mean energy intake and nutrient intake according to the tertiles of ultra-processed food group and unprocessed and minimally processed food were assessed using analysis of covariance and Bonferroni posthoc test. Linear regression analyses were used to identify the direction and statistical significance of the association between tertiles of the energetic contribution of the food groups evaluated and energy and nutrient intake. Both analyses were controlled by confounding factors (age, sex and HVI). The residues were evaluated according to the assumptions of normality, homoscedasticity, linearity and independence.

Collected data were analysed using the Stata^®^ version 11 and a significance level of 5 %.

## Results

A total of 797 children with a mean age of 9·8 ± 0·59 years were evaluated, from which 50·9 % was female and 31 % overweight. It was observed that 16·4 %, 35·4 % and 48·2 % presented low, medium and high/very high HVI, respectively.

Mean energy intake was 8·58 (4·04) MJ, 25·8 % from ultra-processed foods, 56·7 % from natural or minimally processed foods, 8·9 % from culinary ingredients and 8·6 % from processed foods. Industrialised pasta, industrialised cookies, sausages, chocolate powder and soft drinks were the ultra-processed foods most consumed.

Association between energy, macro and micronutrients intake according to the tertiles of ultra-processed foods is presented in Table 1. A decrease in fibre and protein intake was identified, as well as an increase in Na, with the increase of ultra-processed food tertiles (*P* < 0·05). School children in the second and third tertiles presented lower intake of Fe and Zn and higher intake of lipid (*P* < 0·05) compared with the ones in the first tertile. Individuals in the highest tertile of ultra-processed showed a higher energy intake than those in the lowest tertile and also had a lower intake of vitamins A and C (*P* < 0·05).

**Table 1:** Energy and nutrient intake of school children according to tertiles of ultra-processed foods. Belo Horizonte, 2014–2015

	Tertiles of ultra-processed food consumption							
	1st Tertile (≤ 17·58 %)		2nd Tertile (≥ 17·58 % and ≤ 30·26 %)		3rd Tertile (≥ 30·26 %)			
	Adjusted mean	IC 95 %	Adjusted mean	IC 95 %	Adjusted mean	IC 95 %	*P* *	*β* †
Energy (MJ)	8·00^a^	7·48, 8·51	8·69^ab^	8·18, 9·20	8·95^b^	8·44, 9·47	0·028	114·54
Carbohydrate (%)	53·50	52·47, 54·52	52·49	51·48, 53·51	54·02	53·00, 55·04	0·107	0·26
Protein (%)	18·16^a^	17·66, 18·66	16·83^b^	16·34, 17·33	14·78^c^	14·28, 15·28	< 0·001	−1·69
Lipid (%)	28·7^a^	27·88, 29·51	31·15^b^	30·35, 31·95	31·91^b^	31·11, 32·73	< 0·001	1·61
Fibre (g/4·18 MJ)	12·61^a^	12·20, 13·03	11·01^b^	10·60, 11·42	9·73^c^	9·32, 10·14	< 0·001	−1·44
Vitamin A (µg/4·18 MJ)	314·05^a^	256·80, 371·30	245·33^a^	188·83, 301·83	204·02^b^	146·87, 261·17	0·027	−55·01
Vitamin C (mg/4·18 MJ)	49·96^ab^	32·50, 67·42	77·11^a^	59·52, 94·71	39·90^b^	21·62, 58·19	0·012	−4·46
Na (mg/4·18 MJ)	563·39^a^	524·85, 601·93	669·59^b^	631·56, 707·93	740·65^c^	702·18, 779·12	< 0·001	88·62
Ca (mg/4·18 MJ)	324·58	307·76, 341·40	334·73	318·13, 351·34	335·91	319·11, 352·70	0·589	5·66
Fe (mg/4·18 MJ)	6·73^a^	6·52, 6·93	6·04^b^	5·84, 6·25	5·86^b^	5·66, 6·07	< 0·001	−0·43
Zn (mg/4·18 MJ)	6·23^a^	6·04, 6·43	5·68^b^	5·49, 5·87	5·35^b^	5·15, 5·54	< 0·001	−0·44

* ANCOVA with Bonferroni correction; adjustment for age, sex and Health Vulnerability Index.

† Non-standardised Beta coefficients of the linear regression analyses adjusted for age, sex and Health Vulnerability Index.

Mean followed by common letters in the same row are not statistically different (*P* > 0·05).

Linear regression models corroborate these results, except for vitamin C. There was a negative association between consumption of ultra-processed foods and protein, fibre, vitamin A, Fe and Zn and a positive correlation between these foods and caloric, lipid and Na consumption (Table 1).

Table 2 presents the association between energy, macro and micronutrients intake according to the tertiles of unprocessed or minimally processed foods. School children in the highest tertile showed higher intake of protein, fibre, Zn, Fe and lower intake of energy, lipids and Na (*P* < 0·05). Linear regression model (Table 2) confirms the results.

**Table 2:** Energy and nutrient intake of school children according to tertiles of unprocessed and minimally processed foods. Belo Horizonte, 2014–2015

	Tertile of food group consumption							
	First Tertile (< 49·2 %)		Second Tertile (≥ 49·2 % and ≤ 62·2 %)		Third Tertile (≥ 62·21 %)			
	Adjusted mean	IC 95 %	Adjusted mean	IC 95 %	Adjusted mean	IC 95 %	*P* *	*β* †
Energy (MJ)	9·15^a^	8·65, 9·65	8·63^ab^	8·13, 9·14	7·89^b^	7·38, 8·40	0·002	−152·66
Carbohydrate (%)	53·90	52·92, 54·88	53·94	52·94, 54·94	52·70	51·72, 53·73	0·162	−0·56
Protein (%)	14·05^a^	13·61, 14·49	16·59^b^	16·14, 17·03	19·08^c^	18·63, 19·53	< 0·001	2·53
Lipid (%)	32·71^a^	31·94, 33·49	30·03^b^	29·25, 30·82	28·52^c^	27·72, 29·31	< 0·001	−2·12
Fibre (g/4·18 MJ)	9·03^a^	8·66, 9·40	11·49^b^	11·11, 11·87	13·10^c^	12·72, 13·48	< 0·001	2·04
Vitamin A (µg/4·18 MJ)	220·80	169·33, 272·27	267·07	214·89, 319·26	258·90	206·30, 311, 50	0·417	18·63
Vitamin C (mg/4·18 MJ)	33·35	25·05, 41·67	39·78	31·34, 48·22	34·67	26·16, 43·17	0·533	0·75
Na (mg/4·18 MJ)	832·90^a^	798·92, 866·87	661·88^b^	627·43, 696·32	477·79^c^	443·07, 512·51	< 0·001	−177·01
Ca (mg/4·18 MJ)	328·62	312·53, 344·73	342·70	326·36, 359·03	320·06	303·60, 336·53	0·154	−3·29
Fe (mg/4·18 MJ)	5·26^a^	5·08, 5·45	6·28^b^	6·09, 6·47	7·18^c^	6·98, 7·37	< 0·001	0·95
Zn (mg/4·18 MJ)	5·28^a^	5·09, 5·47	5·71^b^	5·51, 5·90	6·28^c^	6·09, 6·48	< 0·001	0·50

* ANCOVA with Bonferroni correction; adjustment for age, sex and Health Vulnerability Index.

† Non-standardised Beta coefficients of the linear regression analyses adjusted for age, sex and Health Vulnerability Index.

Mean followed by common letters in the same row are not statistically different (*P* > 0·05).

## Discussion

Our results found an association between ultra-processed foods consumption and an unfavourable nutrient intake profile, with higher intake of energy, lipids and Na and a lower intake of protein, Fe, Zn and vitamin A.

Similar findings were identified by studies based on household food acquisition in Brazil^(14–16)^ and studies in other countries that verified lower nutritional quality of ultra-processed foods compared with all other food groups^(26,27)^.

Fe, Zn and vitamin A are important nutrients for child development; however, such nutrients are poorly found in ultra-processed foods when compared with unprocessed or minimally processed foods^(10–12)^. A study conducted in Canada with 33 694 individuals aged 2 years and above reported an inverse relationship between ultra-processed foods consumption and the dietary content in protein, fibre, vitamins A, C, D, B_6_ and B_12_, niacin, thiamine, riboflavin, Zn, Fe, Mg, Ca, phosphorus and potassium^(26)^. Similar results were found in the USA^(27)^. The authors suggested that a regular consumption of whole grains, beans, fruits and vegetables, with a reduced participation of ultra-processed food favours a better nutrient intake profile^(26,27)^.

Such results are relevant considering vitamins and minerals are essential in cell signalling, hormone production, immune responses, growth and development. Although micronutrient deficiency does not always manifest clinically, subclinical deficiencies can cause damage to health during childhood^(28)^. Also, the adequate nutrient intake through the effect of food synergy can provide positive effects to child health, especially preventing chronic diseases, which perhaps would not be possible through isolated nutrients^(29)^.

Thus, ultra-processed foods have the potential to increase the risk of obesity, diabetes, CVD and cancer^(30–32)^. Studies conducted in Brazil indicate significant associations of the higher consumption of this food group to metabolic syndrome in adolescents^(33)^, dyslipidaemias in children^(34)^ and obesity at all ages^(35)^.

A previous study reported that a diet rich in ultra-processed foods can contribute to a greater risk of type I diabetes and coeliac disease – important autoimmune disorders in childhood – by inducing intestinal microbiota imbalance and promoting pro-inflammatory response^(36)^. In contrast, a diet based on unprocessed or minimally processed food showed a capacity to promote intestinal microbiota balance, anti-inflammatory response and epithelial integrity^(36)^.

The increased intake of protein, fibre, Zn and Fe and reduced intake of energy fat and Na associated to a higher contribution of unprocessed or minimally processed foods show that the recommendation to always prefer unprocessed or minimally processed foods over ultra-processed foods, preconised by the Brazilian Dietary Guideline^(3,37)^, promotes an adequate and healthy diet. Also, recent study reported that a nutritional intervention using NOVA food groups is effective^(38)^. According to the authors, NOVA is easily understood and applied, therefore, education strategies using food processing knowledge may be effective in the context of the modern food environment^(37)^.

Considering the recall bias, the evaluation of dietary intake through 24hR might not necessarily reflect participant’s dietary habit. However, numerous other studies have used this method for the assessment of food consumption among school children^(33,39,40)^. Also, the study sample consisted of only public-school students of one Brazilian metropolis and almost half of the sample were from high/very high HVI, which can lead to socio-economic homogeneity. Despite the limitations addressed above, the current study stands out for being an investigation regarding a recent field, with a representative sample of school children.

## Conclusion

In conclusion, higher ultra-processed food consumption presented a negative association with the nutrient intake profile of school children. This result highlights the importance of promoting healthy eating habits through food and nutrition education for children, caregivers and the entire school community, attending the current recommendation of prioritising unprocessed and minimally processed foods, moderating processed foods and processed culinary ingredients and avoiding of ultra-processed foods.
